# Survival Analysis, Long-Term Outcomes, and Percentage of Recovery up to 8 Years Post-Infection among the Houston West Nile Virus Cohort

**DOI:** 10.1371/journal.pone.0102953

**Published:** 2014-07-23

**Authors:** Kristy O. Murray, Melissa N. Garcia, Mohammad H. Rahbar, Diana Martinez, Salma A. Khuwaja, Raouf R. Arafat, Susan Rossmann

**Affiliations:** 1 Baylor College of Medicine, Department of Pediatrics, National School of Tropical Medicine, Houston, Texas, United States of America; 2 The University of Texas Health Science Center at Houston, School of Public Health, Houston, Texas, United States of America; 3 Harris County Public Health and Environmental Services, Houston, Texas, United States of America; 4 City of Houston Department of Health and Human Services, Houston, Texas, United States of America; 5 Gulf Coast Regional Blood Center, Houston, Texas, United States of America; Fondazione IRCCS Policlinico San Matteo, Italy

## Abstract

In 2012, we witnessed a resurgence of West Nile virus (WNV) in the United States, with the largest outbreak of human cases reported since 2003. WNV is now endemic and will continue to produce epidemics over time, therefore defining the long-term consequences of WNV infection is critical. Over a period of eight years, we prospectively followed a cohort of 157 WNV-infected subjects in the Houston metropolitan area to observe recovery over time and define the long-term clinical outcomes. We used survival analysis techniques to determine percentage of recovery over time and the effects of demographic and co-morbid conditions on recovery. We found that 40% of study participants continued to experience symptoms related to their WNV infection up to 8 years later. Having a clinical presentation of encephalitis and being over age 50 were significantly associated with prolonged or poor recovery over time. Since the health and economic impact as a result of prolonged recovery, continued morbidity, and related disability is likely substantial in those infected with WNV, future research should be aimed at developing effective vaccines to prevent illness and novel therapeutics to minimize morbidity, mortality, and long-term complications from infection.

## Introduction

West Nile virus (WNV) was first detected in the United States in 1999 [1] and has resulted in a tremendous impact on human health. Since 1999, more than 37,000 human cases of clinical disease have been reported to CDC, including more than 16,000 cases diagnosed with neuroinvasive disease, characterized as meningitis, encephalitis, and/or acute flaccid paralysis [2]. More than 1,500 fatal cases have been reported, predominately in acute encephalitis cases.

Several published studies have reported numerous physical, mental, and neurological consequences up to two years following WNV infection [3]–[10]. There is an important need to improve our understanding of the long-term clinical outcomes in patients infected with WNV. Unfortunately, longitudinal studies consistently following a large number of patients longer than 2 years have not been reported in the literature. -Over a period of eight years, we prospectively followed a cohort of WNV-infected individuals in the Houston metropolitan area to observe recovery over time and define the long-term clinical outcomes. This paper reports our observed outcomes among study participants, percentage of recovery over time, and the effects of demographic and co-morbid conditions on recovery using survival analysis techniques.

## Methods

### Study Population and Ethics Statement

WNV-positive cases identified through surveillance by the City of Houston and Harris County local health departments or during routine screening for viremia at a local blood bank between 2002 and 2009 were invited to enroll in this study. For clinical cases identified through surveillance, the local city and county health departments abstracted medical records as part of their investigation of a reportable disease. Cases were classified by the local health departments using a case definition derived from Sevjar et al [6] into the following groups: (1) encephalitis/meningoencephalitis/encephalomyelitis, including acute flaccid paralysis [WNE], (2) meningitis [WNM], and (3) viral syndrome with fever [WNF]. Neuroinvasive disease was defined as having either WNE or WNM. For blood donor positive study participants, case classification was based on self-report of WNF or being asymptomatic. No positive blood donors were hospitalized for their infection.

Those subjects willing to participate provided written consent prior to interview. For those study participants who were clinically incapacitated, a surrogate (next of kin or guardian) most familiar with the patient’s activities and clinical illness was identified to provide consent on behalf of the study participant. This study protocol and written consent form was reviewed and approved by Baylor College of Medicine (H-30533) Institutional Review Board and the University of Texas Health Science Center at Houston Committee for the Protection of Human Subjects (HSC-SPH-03-039).

### Data Collection

Study participants enrolled into this study who developed clinical disease during the acute phase of infection were interviewed by phone or in person every six months for up to 8 years post-infection. Study participants were asked if they were continuing to experience symptoms from their WNV infection; however, if the patient reported no further symptoms and a return to their pre-WNV baseline status, then the date of recovery (actual date the interviewee felt they had returned to their baseline status) was documented. To discourage biased reporting of symptoms, study participants were asked using an open ended sentence what symptoms they were still experiencing since their infection with WNV. Study participants were followed and reassessed every six months using the same qualitative observations. Data were used to ascertain clinical outcome, recovery rates, and identify any continuing physical or mental deficits.

### Data Analysis

Follow-up interviews were entered into an Epi-Info 2002/Access database. Data were analyzed using the statistical functions of NCSS (NCSS, Inc; Kaysville, Utah) and STATA 11.0 (STATACORP; College Station, TX). Initial patient interviews and follow-up interviews provided data for analysis. If study participants were blood donors who did not develop symptoms related to their WNV infection, they were excluded from further analysis.

The recovery status of all subjects was assessed every 6 months over the course of the study. The time to recovery was calculated by determining the number of days from onset of symptoms to the exact date the subject reported returning to their baseline level of functioning (level of health before infection with WNV). For study participants who did not report returning to their baseline function during the follow-up period, we considered this a censored observation. Specifically, time to censoring was defined as the number of days from onset of illness to date of the most recent interview or date of death in which the subject or their surrogate reported continuing symptoms related to their WNV infection. The time variable in the survival analysis was defined as the minimum of time to recovery or time to censorship. The survivorship function [S(t) = Prob(time to recovery is longer than *t*)] and percentiles were estimated using the Kaplan-Meier product-limit survival distribution [11]. As a separate analysis, a similar survival technique was used to examine the distribution of time to death in study participants over the course of study. Time to death was defined as onset of symptoms from their WNV infection to the date of death as verified by family members and social security death index. For study participants who did not die over the course of follow-up, we defined censoring time as the number of days between onset of symptoms and either date of recovery or date of last follow-up interview.

Univariate and multivariate Cox regression models were performed to identify demographic, medical, and social history variables that could be associated with poor or prolonged recovery from WNV. Following univariate analysis, variables identified with a p-value≤0.25 were entered into the multivariate Cox regression model. A backward step-wise elimination approach was used to determine the best predictive model for recovery (α = 0.05); however, for inference, we used the probability of a type I error of 0.05. Hazard ratios, p-values, and 95% confidence intervals were calculated.

## Results

A total of 381 WNV positive subjects were identified during 2002–2009 in metropolitan Houston, Texas according to data reported to CDC’s Arbonet [12], including 314 clinical WNV cases and 67 positive viremic blood donors. Of those hospitalized for their infection, 21 (8.2%) of these cases died acutely as a result of their WNV illness. Out of 381 participants eligible for our study, 157 (41%) were available and agreed to participate; 81% of study participants were enrolled within one year of onset of illness. Table 1 presents the demographic characteristics of all study participants. Most (60%) of the study participants were infected between 2002 and 2004. The majority of participants presented with neuroinvasive disease (n = 98, 62%), were male (n = 98, 62%), and white, non-Hispanic (80%). Median age of participants was 54 years, with a range of 9 to 87 years.

**Table 1:** Houston WNV Cohort Demographics reported at the time of onset of illness, 2002–2009.

Patient follow-up findings	No. (%), n = 157
Median age of study participants	54 years (range 9–87)
Males	98 (63)
Race/Ethnicity:	
White, non-Hispanic	125 (80)
Black	19 (12)
Hispanic	10 (6)
Asian	1 (1)
Other/unspecified	2 (1)
Clinical Presentation:	
WNE	68 (43)
WNM	30 (19)
WNF	39 (25)
Asymptomatic	20 (13)
Year of WNV diagnosis:	
2002	46 (29)
2003	31 (20)
2004	18 (11)
2005	16 (10)
2006	26 (17)
2007	13 (8)
2008	2 (1)
2009	5 (3)
Occupation before illness:	
Employed	98 (62)
Retired	34 (22)
Disabled	4 (3)
Student	6 (4)
Homemaker	7 (5)
Unemployed	4 (3)
Hospitalized for WNV illness	118/157 (75)
Discharged home	88/118 (75)
Discharged to rehabilitation/long term care/hospice	18/118 (15)
Discharged to care of family or friends	8/118 (7)
Died in hospital or long-term care/hospice facility	4/118 (3)
Died after hospitalization (over the course of the study)	13/153 (8)

The majority (76%) of study participants who developed clinical disease from their infection were hospitalized. Of those hospitalized, 88 (74%) were discharged home, 18 (15%) were transferred to a long-term care or rehabilitation facility, 8 (7%) were discharged to the home of a family member or friend for care, and 4 (3%) died in hospital.

At one year post-infection, 60% (62/103) of study participants were still reporting symptoms related to their illness from WNV (Table 2). The most commonly patient-reported sequelae were fatigue, weakness, depression, difficulty walking and/or feeling off balance, and memory loss. Paralysis was reported by 9% of study participants, followed by tremors (5%) and seizures (1%). Two years following infection, 47% (44/94) were still reporting symptoms, with the most commonly reported complaints being fatigue, weakness, difficulty walking, depression, and memory loss; 4% of study participants were still reporting paralysis. After five years, 40% (29/73) were still reporting continued symptoms, with 26% of participants reporting depression. By eight years post-infection, 40% (18/45) were still reporting WNV-related sequelae, with fatigue, depression, weakness, and neck/back pain most commonly reported.

**Table 2:** Patient-reported clinical sequelae following symptomatic West Nile virus infection.

Patient reportedclinical sequelae	1 year post-onset(%), n = 103	2 years post-onset(%), n = 94	5 years post-onset(%), n = 73	8 years post-onset(%), n = 45
Number still reporting symptoms	62 (60)	44 (47)	29 (40)	18 (40)
Patient-reported sequelae:				
Fatigue	38 (37)	21 (22)	6 (8)	9 (20)
Weakness	20 (19)	16 (17)	11 (15)	6 (13)
Depression	18 (17)	14 (15)	19 (26)	8 (18)
Difficulty walking/ataxia	15 (15)	15 (16)	6 (8)	2 (4)
Memory loss	11 (11)	11 (12)	5 (7)	4 (9)
Paralysis	9 (9)	4 (4)	3 (4)	0 (0)
Headaches	9 (9)	7 (7)	3 (4)	2 (4)
Joint pain	9 (9)	7 (7)	3 (4)	4 (9)
Neck/back pain	9 (9)	5 (5)	3 (4)	6 (13)
Blurred vision	8 (8)	1 (1)	2 (3)	2 (4)
Muscle pain	7 (7)	5 (5)	1 (1)	3 (7)
Confusion	7 (7)	1 (1)	2 (3)	4 (9)
Weight loss/anorexia	7 (7)	1 (1)	1 (1)	0 (0)
Tremors	5 (5)	2 (2)	1 (1)	0 (0)
Dizziness	5 (5)	3 (3)	3 (4)	0 (0)
Stiff neck	3 (3)	2 (2)	0 (0)	0 (0)
Seizures	1 (1)	1 (1)	0 (0)	0 (0)
Recurring fever	1 (1)	2 (2)	1 (1)	2 (4)
Still seeing physician for sequelae	42 (41)	30 (32)	10 (14)	6 (13)
Cumulative no. of deaths over course of follow-up	0	2	9	13

**Figure 1:**
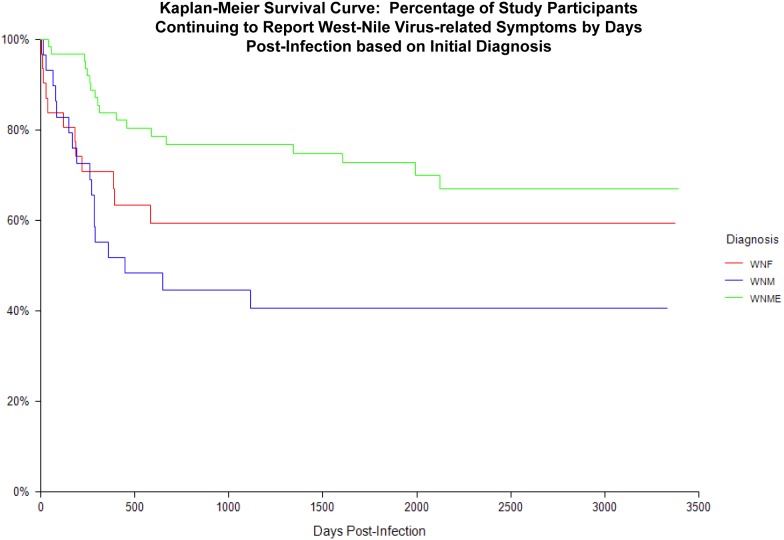
Kaplan-Meier Survival Curve: Percentage of Study Participants Continuing to Report West-Nile Virus-related Symptoms by Days Post-Infection based on Initial Diagnosis.

Based on logrank test (Figure 1), significant differences were seen between groups based on initial diagnosis (chi-square = 9.6; degrees of freedom = 2; p = 0.008), with study participants who presented with WNE having the lowest percentage of recovery to baseline over time. Across all groups, recovery plateaus around 2 years post-infection. At 2000 days (5.5 years) post-infection, 70% of WNE participants were still reporting what they perceived as symptoms related to their WNV infection (95% CI = 57.8% to 82.3%).

**Figure 2:**
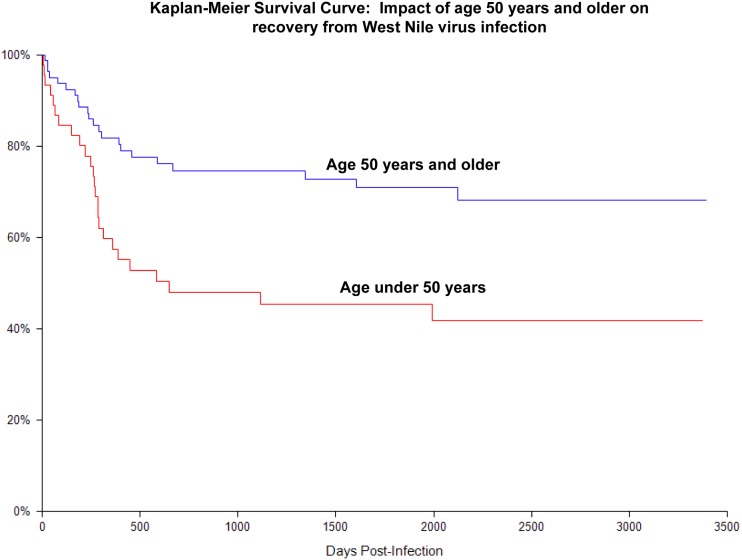
Kaplan-Meier Survival Curve: Impact of Age 50 and older on recovery from West Nile virus infection.

To understand the impact of potential demographic, medical, and social factors on percentage of recovery over time (Table 3), we conducted a univariate Cox regression analysis and identified the following variables for inclusion in the multivariate model: age ≥50 years, race/ethnicity (non-Hispanic, white), history of hypertension, history of diabetes mellitus, chronic alcohol use, and illicit drug use. Based on a final multivariate Cox regression model, age was the only variable found to be independently associated with poor or prolonged recovery, with participants age 50 and older being more likely to have a prolonged recovery (Adjusted Hazard Ratio = 1.96, 95% CI:1.09–3.54; see Kaplan-Meier curve, Figure 2).

**Table 3:** Univariate and multivariate Cox regression analysis results for the impact of demographics, social, and medical co-morbidities on recovery from clinical West Nile virus (WNV) infection (encephalitis, meningitis, and uncomplicated fever).

Demographic,medical and socialhistories priorto infectionwith WNV	All clinicalWNV studyparticipants,n = 129 (%)	Recovered tobaselinestatus,n = 47 (%)	Continuing toreport WNV-relatedsymptoms at lastinterview,n = 82 (%)	Univariate RiskRatio (95% CI);p-value	MultivariateRisk Ratio*(95% CI);p-value
Age≥50 years	83 (64)	22 (47)	61 (74)	2.35 (1.32–4.17); p = 0.004	1.96 (1.09–3.54); p = 0.024
Male	81 (63)	30 (64)	51 (62)	1.09 (0.60–1.98); p = 0.78	
Non-Hispanic, white	99 (77)	36 (77)	63 (77)	2.14 (1.19–3.86); p = 0.011	NS
Hypertension	58 (45)	18 (38)	40 (49)	1.49 (0.82–2.69); p = 0.19	NS
Diabetes mellitus	23 (18)	4 (9)	19 (23)	2.93 (1.05–8.18); p = 0.04	2.42 (0.85–6.87); p = 0.097
Stroke	12 (9)	3 (6)	9 (11)	1.82 (0.56–5.85); p = 0.32	
History of cancer	13 (10)	3 (6)	10 (12)	0.63 (0.20–2.06); p = 0.45	
Chronic alcohol use	14 (11)	8 (17)	6 (7)	0.57 (0.27–1.23); p = 0.15	NS
Chronic tobacco use	67 (52)	23 (49)	44 (54)	1.18 (0.66–2.11); p = 0.57	
Illicit drug use	8 (6)	5 (11)	3 (4)	0.52 (0.22–1.24); p = 0.14	NS

*Variables with p≤0.25 were entered into the multivariate model. A backward-stepwise approach was used to determine independent variables considered significant (α = 0.10).

**Chronic alcohol use was defined as having a history of more than 15 drinks containing alcohol per week.

***NS = Not significant.

**Figure 3:**
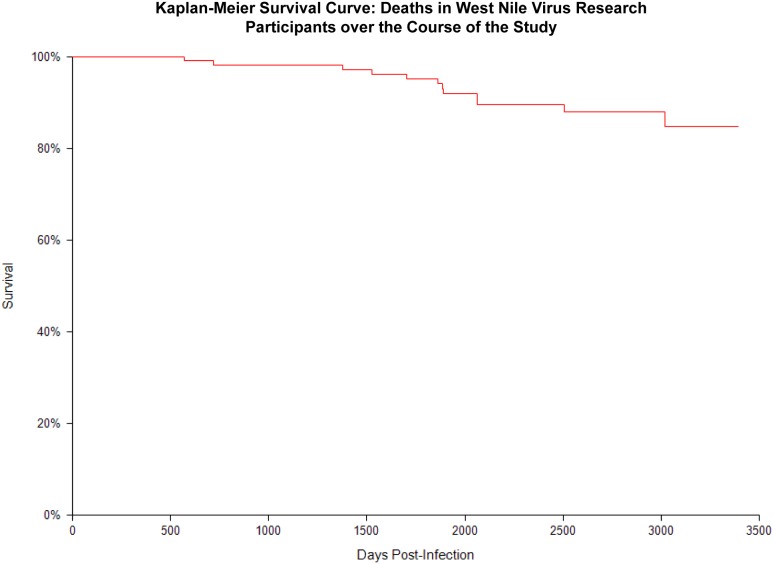
Kaplan-Meier Survival Curve: deaths in West Nile virus research participants over the course of the study.

Thirteen of the 157 study participants (7.6%) died over the course of the study, (Table 2; Figure 3), with time between the date of onset of WNV illness to date of death ranging from 568 days (1.6 years) to 3,018 (8.3 years). Age at time of death ranged from 40 years to 92 years, with a median age of 75 years. Ten (77%) of the 13 deaths were in participants who initially presented with encephalitis, while one presented with meningitis and two others presented as an uncomplicated fever case.

## Discussion

The long-term clinical outcomes, including any prolonged or permanent mental or physical deficits, in patients infected with WNV critically needs to be defined. This study is the first to prospectively follow a large number of individuals infected with WNV for an extensive period of time, and the first to use survival analysis techniques to examine rates of recovery and identify factors that could affect recovery. Based on the results of this study, we found that WNV infection can result in continued morbidity, with 40% of study participants reporting what they perceive to be symptoms related to their initial infection up to 8 years later. Using survival analysis techniques, we found that recovery plateaued around 2 years post-infection. Those participants who initially presented with encephalitis were significantly more likely to have poorer outcomes, with 70% still reporting continued symptoms more than 5 years following their initial illness. Interestingly, those who initially presented with uncomplicated fever appeared to have poorer outcomes compared to those who presented with WNV meningitis. In addition to severity of disease at the time of acute disease, being over 50 years of age was also significantly associated with poorer clinical outcome following WNV infection.

One important strength of this study is that we had the opportunity to prospectively follow a large cohort of WNV-positive subjects over such an extensive period of time. Other studies published to date focused on clinical outcomes in the first year or two following infection and typically involved smaller sample sizes. One study of hospitalized patients who were infected with WNV in New York City in 1999 found that 63% of 35 patients reporting continued symptoms up to a year following infection, with most reporting muscle weakness, loss of concentration, confusion, and lightheadedness [4]. Most of the patients in this study were 65 and older (63%) and presented with encephalitis (54%). Similar to our study, they found that age at the time of infection was a significant predictor of recovery. In Tennessee, 55% of 22 patients were still reporting complications from their illness one-year post-infection, with fatigue, weakness, difficulty ambulating, and memory difficulties most commonly reported [5]. In North Dakota, 49 patients were assessed at around 13 months post-infection, and long-term morbidity was also reported, including symptoms of fatigue, memory impairment, weakness, headache, joint pain, and balance problems [3]. Following the 2002 outbreak in St. Tammany Parish, Louisiana, 16 patients with neuroinvasive WNV infection were re-examined eight months post-infection to assess symptoms, functional status, and neurologic sequelae [6]. In this study, patients continued to report fatigue, headache, myalgias, gait and movement disorders, and neurological weakness. Compared to the New York, Tennessee, North Dakota, and Louisiana studies, our findings in Houston were comparable at the one year post-infection assessment in that 60% of our study participants were reporting similar complications.

In contract, a study by Loeb et al. [10] followed 156 Canadian patients up to two years following infection, and the investigators found that physical and mental function normalized in most of the patients by one year post-infection, with neuroinvasive disease cases and those with pre-existing co-morbidities being statistically more likely to take a longer period of time to recover. One of the main differences between our study and the one by Loeb et al was the proportion of non-neuroinvasive disease cases (28% of symptomatic cases in our cohort compared to 59% of cases in the Canadian study). Another difference was the use of only objective measurements (physical and mental functioning) in the Canadian study, compared to our use of patient-reported outcomes. While the objective measures do provide a more quantifiable approach, our capturing of patient-reported outcomes allowed us the opportunity to understand the high prevalence of chronic somatic complaints following infection.

We were not expecting to find that study participants who initially presented with meningitis to report better recovery than those who initially were diagnosed with uncomplicated fever. It is possible that those with the fever presentation were misclassified since most (80%) participants were severely ill enough to seek medical care from a hospital emergency center, and 75% of those individuals were admitted, with a median hospitalization stay of 8 days. It is possible that in fact these were neuroinvasive disease cases, but were not classified as such because there was no analysis of cerebrospinal fluid (CSF). One of our defining criteria for WNV meningitis or encephalitis is to have evidence of pleocytosis in the CSF. If no lumbar puncture procedure was performed, or if the tap was contaminated with blood, then they could not be evaluated for or classified as a neuroinvasive disease case. Interestingly, the median age of WNF cases was somewhat older than WNM cases (52 years versus 46 years, respectively), while the median age of WNE cases was 63 years. Being that WNF cases were older, they would be more likely to have other co-morbid conditions that could contribute to their hospitalization and recovery over time. Regardless, delayed and poor recovery in those with presumed milder disease warrants further investigation. One study by Carson et al. also found high rates of somatic complaints in non-hospitalized fever patients and clinical abnormalities equivalent to those who were hospitalized with neuroinvasive disease [3]. The authors postulated that the WNV fever presentation might actually be subclinical encephalitis that results in pathological changes that are not necessarily benign.

The Houston WNV cohort has been a valuable resource for understanding clinical outcomes following infection. As reported here, depression was a common complaint among study participants. Beginning in 2003, we began to implement the Center for Epidemiological Studies Depression Scale (CES-D) to better quantify depression among our cohort participants [13], [14]. In a one-year follow-up study, we found that 31% of study participants were reporting new onset depression following their infection with WNV; 75% of these individuals had CES-D scores indicative of clinical depression [13]. In a follow-up study, we found that depression continued to be reported up to 8 years post-infection with CES-D scores again being supportive of clinical depression [14]. As part of the larger study, we are also monitoring kidney function in our participants since we have identified an overall prevalence of chronic kidney disease to be 40% [15]. Finally, over the course of our study, 4 individuals died during their initial hospitalization for WNV, and 12 participants died over the course of follow-up. One study out of Colorado reported a two-fold increase in mortality up to 3 years post-infection [16]. We have initiated a study to examine whether WNV-positive individuals in our study are also at increased risk for mortality when compared to the general population.

Limitations of this study that are important to discuss are the possibility of selection bias and interviewer bias. This study attempted to contact and enroll all WNV-positive individuals identified through health department surveillance and blood bank screening, however, the racial/ethnic backgrounds of study participants was different from the Houston census population [17], with an over-representation of non-Hispanic whites (80% vs. 32.2%), and an under-representation of blacks (12% vs. 19.5%) and Hispanics (6% vs. 41.5%). Previous reports on WNV outbreaks in Houston [18] and Texas [19] found the clinical attack rates to be highest in non-Hispanic whites and lowest in Hispanic populations; however, we still feel based on our attempts that we had under-representation of minority groups. Spanish speaking interviewers and introduction of the use of compensation for time involved in the study were used to try and maximize participation, however the response rate overall was still disappointing below par (41%), with most non-enrolled patients being lost to follow-up following their hospitalization. For those who were enrolled, we were impressed by their willingness to remain committed to the study for such an extensive period of time.

Another important limitation to mention is the generalizability of this study. With WNV infection, only roughly 20% of people are symptomatic, with less than 1% presenting with neuroinvasive disease [20]. In our study, 62% of participants had neuroinvasive disease. This is similar to surveillance findings in Texas, whereby 67% of cases reported between 2002 and 2011 were neuroinvasive disease cases [21]. While we made every attempt to enroll all cases identified through surveillance, it is very possible that those who chose to participate and continued to participate were more likely to have experienced severe disease and were concerned about sequelae. At the time of the 8 year follow-up, we were only able to include cases from 2002 and 2003. Out of 71 symptomatic cases who were infected in those years and enrolled at the beginning of the study, only 45 (63%) were available for the 8 year follow-up visit. Of the 26 not represented, 9 had died, and the remaining had either moved away from the area (n = 5), were lost to follow-up (n = 9), unavailable at the time of the follow-up (n = 2), or refused to participate (n = 1). With a decrease in the number of participants over time, the results have the potential to be less reliable due to decreased representativeness and increased variance. While this could be an influential bias, we still felt it was important to report the long term experiences of these individuals and identify factors that could impede recovery. Finally, since the patients were responsible for reporting their symptoms that they felt were related to their initial infection, it is possible that these complaints could be a result of some other condition. We are in the process of conducting a matched case-control study to examine these findings as they relate to a comparison group of the same age, gender, and race/ethnicity.

## Conclusions

In 2012, we witnessed a resurgence of WNV in the United States, with the largest outbreak of human cases reported since 2003 [2], [19]. In Texas alone, we had more than twice the number of reported cases than the historical high in 2003, with more than 1,800 cases of clinical WNV reported, including 89 deaths [19]. WNV is now endemic and will continue to produce epidemics over time, therefore defining the long-term consequences of WNV infection is critical. Based on our findings, WNV infected patients who are over age 50 and/or initially presented with encephalitis could be at higher risk for delayed or poor recovery. Even though we did not find diabetes to remain significant in the multivariate cox regression analysis in this study (p = 0.097), diabetes could also be a potential factor that can impede the recovery process. Since the health and economic impact as a result of prolonged recovery, continued morbidity, and related disability is likely substantial in those infected with WNV, future research should be aimed at developing effective vaccines to prevent illness and novel therapeutics to minimize morbidity, mortality, and long-term complications from infection.
